# Evaluating buprestid preference and sampling efficiency of the digger wasp, *Cerceris fumipennis,* using morphometric predictors

**DOI:** 10.1093/jis/14.1.4

**Published:** 2014-01-01

**Authors:** Warren E. Hellman, Melissa K. Fierke

**Affiliations:** 1 Washington State Department of Agriculture, 1111 Washington Ave, Olympia, WA 98501; 2 State University of New York College of Environmental Science and Forestry, 1 Forestry Drive, Syracuse, NY 13210

**Keywords:** biomonitoring, biosurveillance, Crabronidae, insect museum collections, nest provisioning, wasp foraging behavior

## Abstract

In-ground colonies of the native digger wasp, *Cerceris fumipennis* Say (Hymenoptera: Crabronidae), were sampled over two years in four New York State counties to characterize prey range, primarily their preying on beetles in the metallic wood-boring family, Buprestidae. These records were also used to evaluate beetle sampling efficiency by comparing collected beetles to historic county records and to identify limitations of wasp-mediated sampling in study areas. Overall, 1,530 beetles representing three families and 44 beetle species were collected from *C. fumipennis* . Five of these species ( *Agrilus cuprescens* (Ménétriés) (Coleoptera: Buprestidae), *A. pensus* Horn *, Buprestis nutalli* Kirby *, Chrysobothris scabripennis* Gory and Laporte *, Dicerca pugionata* (Germar)) were new prey records for *C. fumipennis* . The wasps exhibited a strong preference for larger beetle genera (e.g., *Dicerca, Buprestis* ), which accounted for 68% of beetles caught. *Agrilus* and *Chrysobothris* were the next dominant genera, accounting for 16% and 11%, respectively. A 4–19 mm prey size range is proposed, as all beetles collected were within this range despite the availability of prey outside of this range. *Cerceris fumipennis* caught 43% of the 42 buprestids species present in museum records from the four census counties as well as an additional 23 buprestid species that were not represented in museum records. Of the 22 buprestid species identified in museum collections that were not caught by *C. fumipennis* in the census counties, only one was within the proposed size range and active during the *C. fumipennis* flight season (late June through August). Overall, sampling *C. fumipennis* colonies over two summers at five sites resulted in 32% of the recorded buprestid species in New York State being caught, indicating that monitoring colonies is an efficient and viable means of quantifying buprestid assemblages.

## Introduction

There are nearly 15,000 known species of jewel beetles (Coleoptera: Buprestidae) worldwide, with 787 species occurring in Canada and the United States ( Nelson et al. 2008 ). Most buprestids feed in stressed, dying, or dead trees though some introduced species, e.g., the emerald ash borer, *Agrilus planipennis* Fairmaire, which can attack and kill healthy trees ( Haack et al. 2002 ; Cappaert et al. 2005). Only a few buprestid species are considered economically injurious in the United States, e.g., the two-lined chestnut borer, *A. bilineatus* Weber, and the bronze birch borer, *A. anxius* Gory; therefore, relatively few taxa have been closely studied ( Carlson and Knight 1969 ). The life history of *A. planipennis* makes studying them problematic and labor intensive; larvae are cryptic, hidden within leaves, or under bark during development, and most adult stages rest, feed, and mate in tree canopies out of reach of conventional trapping methods ( Timms et al. 2006 ).

The most common detection method for adult wood-boring beetles has been the use of baited (chemical lures) or unbaited passive flight intercept traps ( Chénier and Philogène 1989 ; Leather 2005 ). Historically, however, these methods result in relatively low yields of buprestids ( Chénier and Philogène 1989 ; McIntosh et al. 2001 ; De Groot and Nott 2003 ). Recently, *Cerceris fumipennis* Say (Hymenoptera: Crabronidae), a native digger wasp, was suggested as a taxa-specific monitoring tool for detection of Buprestidae ( Marshall et al. 2005 ). Female *C. fumipennis* provision their larvae almost exclusively with paralyzed buprestids in multiple cells within their subterranean nests. The buprestid prey can be collected by intercepting females as they return to their nests. Monitoring *C. fumipennis* nests has been shown to be an efficient way to collect buprestids and is being proposed as an alternative detection tool for *A. planipennis* at low densities ( Careless 2009 ).

Consideration of *C. fumipennis* as a sampling tool requires an understanding of foraging behavior, including factors influencing prey selection. *Cerceris fumipennis* are known to prey on nearly 90 species of Buprestidae across 12 genera from all four North American subfamilies ( Careless 2009 ). Despite their selectivity for buprestids, prey vary in appearance (e.g., shape, color, metallic luster) with no apparent uniformity at the family level to explain prey choice. Chemical assays are being undertaken to search for non-visual cues for prey location. Curiously, *C. fumipennis* is known to infrequently take anomalous (nonbuprestid) prey. Documented nonbuprestid prey includes small Chrysomelidae ( *Neochlamisus bebbinae* Brown *, Bassareus mammifer* Newman, *Leptinotarsus decemlineata* Say), mid-sized Scarabaeidae ( *Popilia japonica* Newman), and Cerambycidae ( *Saperda discoidea* F. *, Oberea schaumii* LeConte) ( Rutledge et al. 2011 ). As more *C. fumipennis* colonies are observed, it is becoming apparent that capture of these apparently anomalous prey are not isolated incidents and our understanding of prey selection is far from complete.

The overall goal of this study was to census New York Buprestidae using *C. fumipennis* as a sampling tool. Specific objectives were to: 1) identify beetles targeted by *C. fumipennis* , 2) compare how catches differed among colonies, 3) elucidate size range of prey, and 4) evaluate sampling efficiency of *C. fumipennis* relative to historical (i.e., museum) collections.

## Materials and Methods

### Site descriptions.

**Figure 1.**
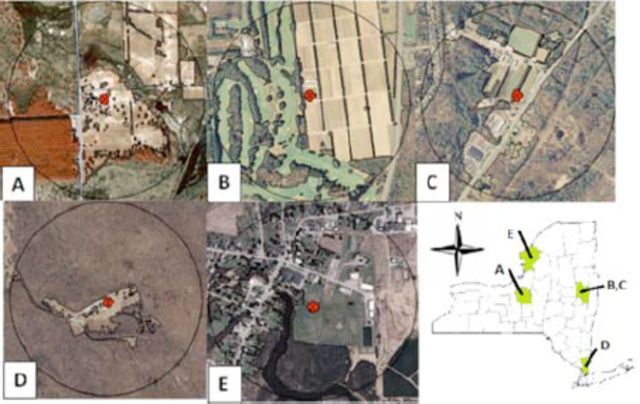
Aerial images of five *Cerceris fumipennis* colonies within a 0.5 km radius. (A = Three Rivers Wildlife Management Area, B = RT9, C = RT50, D = WPR, E = LaFargeville Central School). High quality figures are available online.

Five long-term study sites were selected in New York State at locations where *C. fumipennis* nests were concentrated into small colonies ( Table 1 , Figure 1 ). The number of active nests in a colony varied daily and seasonally but were only sampled when > 30 nests were present. All study sites had sandy loam soils, received direct sun, and were near patchy forest.

**Table 1.**
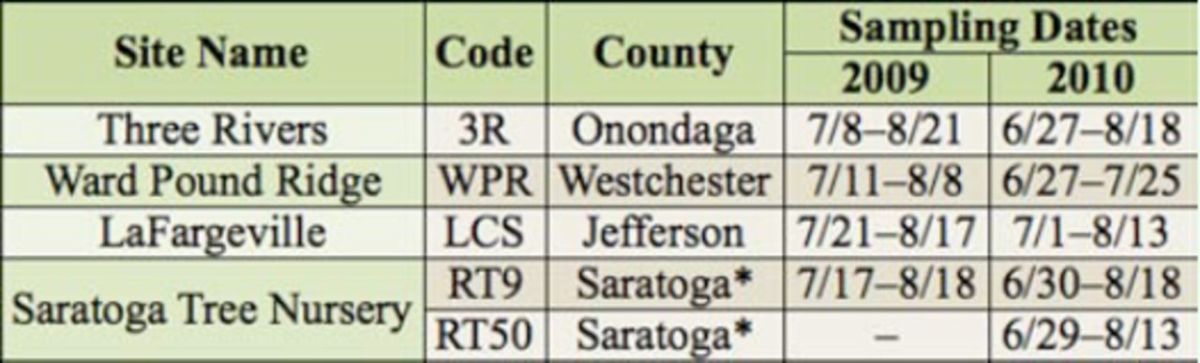
*Cerceris fumipennis* colony locations and sampling dates for Buprestidae sampling in four New York counties.

*Two colonies were sampled in Saratoga County

Three Rivers Wildlife Management Area is a New York State Department of Environmental Conservation wilderness area just north of Baldwinsville in Onondaga County. *Cerceris fumipennis* nests are located along a seldom-used sandy access road and in a sandy area approximately 50 m west of the road ( Figure 1A ). The site is composed of mowed fields with fragmented clumps of hardwood and conifer trees. Saratoga Tree Nursery properties are located in Saratoga County and are managed by New York State Department of Enviromental Conservation. One monitored *C. fumipennis* colony was off of Route 9 (RT9) in the outskirts of Saratoga Springs, and one was bordered by a golf course and naturally wooded areas. Nests occurred in an open sandy road on the property ( Figure 1B ). The Route 50 (RT50) colony was 2.7 km southwest of the RT9 colony. It was situated alongside a highway in a mix of conifer and hardwood forest and nursery trees ( Figure 1C ). Ward Pound Ridge County Park (WPR) is a multi-use recreation area in Westchester County. The *C. fumipennis* colony was located in an un-paved, overflow parking area with many large open grown trees and was surrounded by a large hardwood forest ( Figure 1D ). A large *C. fumipennis* colony was located on the LaFargeville Central School baseball field in Jefferson County ( Figure 1E ). The baseball diamond is part of a larger athletic field and is bordered on two sides by a forested wetland. Beyond the athletic field, the immediate area consists of mostly residential homes and farmland.

### Beetle collection

Beetle collection was carried out via two methods: 1) intercepting female C *. fumipennis* as they returned to their nests with prey and 2) locating paralyzed buprestids dropped by *C. fumipennis* on the ground near C *. fumipennis* nests. Interception of beetle-carrying *C. fumipennis* caused them to release their prey, resulting in the collection of live beetles. This was accomplished by net-ting wasps midflight or antagonizing them either in-flight or just prior to them entering their nests. Prey-laden *C. fumipennis* were visually recognized by profile and flight pattern. *Cerceris fumipennis* with prey have a discernable bulge below the thorax and fly more directly toward their nest with slower, wider turns compared to unburdened *C. fumipennis* , whose flight pattern is more erratic.

To facilitate simultaneous monitoring of numerous nests, small ‘collars’ were placed over nest entrances ( Careless 2009 ). A collar is a piece of plastic or cardstock with a hole punched in it that is aligned with the nest entrance. Collars were anchored using a golf tee or by placing sand or a small rock on a corner. The small diameter of the hole allowed an unburdened female wasp to enter and exit the nest but inhibited her from entering with prey.

Collection of beetles from *C. fumipennis* and dropped beetles occurred every monitoring day. Beetles were collected over 2–7 hrs or until 30–50 beetles were obtained. This beetle quota was obtained from rarefaction curves derived by Careless (2008) , who demonstrated that 30–50 beetles provided a robust number of species with a sharply decreasing likelihood of encountering unrecorded species beyond this range.

Three Rivers Wildlife Management Area and WPR colonies were sampled weekly, beginning the first week of adult emergence (8 July in 2009 and 27 June in 2010) and lasting until the number of active *C. fumipennis* nests fell below the threshold of 30. In 2009, RT9 and LaFargeville Central School were sampled only three times each during the flight season due to travel distances for the researchers. RT50 was not sampled in 2009, but was sampled weekly in 2010. Some weeks it was not possible to collect the beetle quota due to unfavorable weather.

### Beetle identification

Collected specimens were identified using Bright (1987) . Specimens from the 2009 season at WPR were identified by Dr. Claire Rutledge at the Connecticut Agricultural Experiment Station, and ambiguous specimens were sent to E. Richard Hoebeke for confirmation. All other specimens were identified by W. Hellman and confirmed by E. Richard Hoebeke at Cornell University. Voucher specimens are housed in the State University of New York College of Environmental Science and Forestry Insect Museum.

### Museum collections

A Buprestidae species record for censused counties was compiled using collection specimens from Cornell University in Ithaca, NY; New York State Museum in Albany; American Museum of Natural History in New York City; and State University of New York College of Environmental Science and Forestry in Syracuse. Museum records dated back over a century and provided a large composite source of buprestid distribution records. At each museum collection, Buprestidae holdings were reviewed, noting the date and location for each record from censused counties. Collection dates were used to estimate periods of adult beetle activity for specific species.

### Determining prey range

Morphometric ranges of prey were measured for prey items and museum specimens. Whole body length and thoracic width measurements were taken using digital cali-pers from 62 beetle species. Some beetles collected by *C. fumipennis* and from museums were not measured due to specimen damage and museum curator’s handling restrictions. Depending on the number of available specimens, up to 10 individuals from each species were measured to generate average dimensions. For several species, only one specimen was available. Specimens were arbitrarily selected for measurement after counting the number of available beetles (N), dividing by ten, and measuring every *n* th specimen as determined by the quotient ( *n* = N/10). Regression analysis of buprestid length against width revealed length was a good predictor of thoracic width (R ^2^ = 0.88; *p* < 0.0001), and so length measurements are used to represent relative size.

### Prey choice

Head capsule width was measured for 46 female *C. fumipennis* returning with prey to quantify prey choice at the Saratoga *C. fumipennis* colonies (RT9, RT50) from 22 July to 17 August 2010. Wasps were subdi-vided into three categories by head size (small: 3.2–4.0 mm; medium: 4.1–4.9 mm; large: 5.0–5.8 mm). Corresponding prey were identified to genus and divided into two size categories (small, e.g., *Agrilus* , *Chrysobothris* ; large, e.g., *Dicerca* , *Buprestis* ) based on average length measurements for each genus as described above. *Dicerca* (15.9 ± 0.3 mm, n = 42) and *Buprestis* (15.4 ± 0.4 mm, n = 29) species are on average much larger than *Chrysobothris* (8.7 ± 0.3 mm, n = 47) and *Agrilus* (7.4 ± 0.2 mm, n = 79) beetle species (F1,196 = 788, *p* = < 0.0001). Prey mass was also measured for the five largest and smallest beetles (based on the length measurement) caught on 22 July 2010. These measurements were used to estimate upper and lower weights of prey items that *C. fumipennis* captured.

### Data analysis

A likelihood-ratio (G ^2^ ) analysis of wasp size and prey size was used to evaluate prey size bias in *C. fumipennis* foraging behavior. Species richness was the total number of beetle species identified at each site, and beetle diversity was calculated using the Shannon-Wiener index (H’ = - Σpi(ln pi) where pi = the proportion of individuals of species i). All data analyses were completed in JMP8 ( SAS 2009 ).

## Results

### Beetle Sampling

Overall, 1,530 beetles representing 44 species from 13 genera and three families were collected from *C. fumipennis* during the two field seasons ( Table 2 ). In 2009, 654 beetle captures yielded 32 species of Buprestidae and two species of Chrysomelidae. More intensive weekly sampling in 2010 at all five colonies yielded 876 specimens representing 35 species of Buprestidae, two species of Chrysomelidae, and one species of Cerambycidae.

**Table 2.**
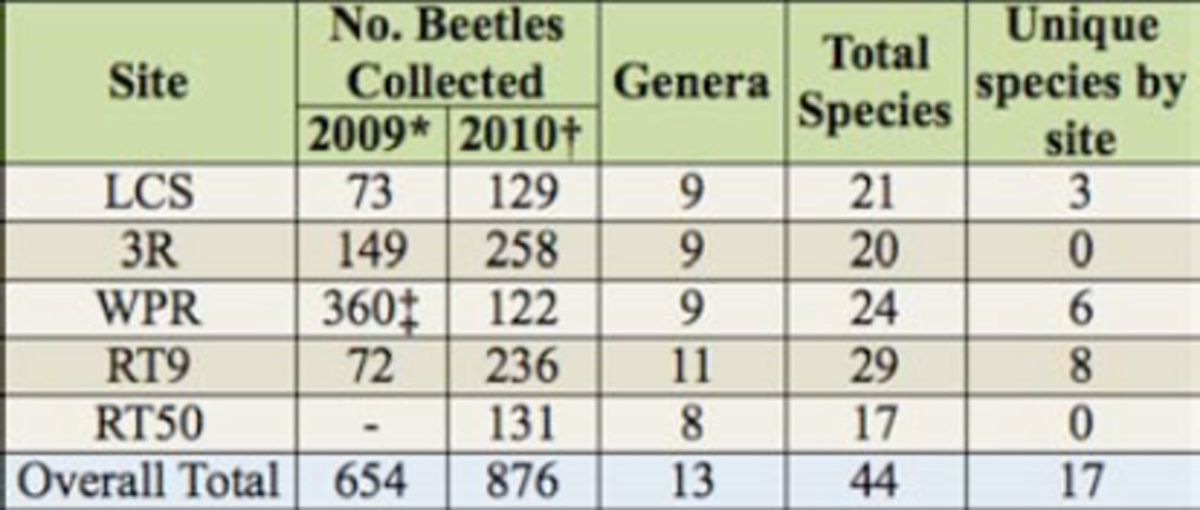
Summary data by site for beetles collected at New York *Cerceris fumipennis* colonies in 2009 and 2010.

*In 2009, colonies were monitored weekly at Three Rivers Wildlife Management Area and WPR and three times at LaFargeville Central School and RT9.

†In 2010, all colonies were monitored weekly.

‡WPR colony was monitored more frequently than weekly in 2009.

Ten additional beetle species were captured in 2010 that were not collected in 2009 despite considerable overlap of species capture. Over the two years of study, five new prey species for *C. fumipennis* in the family Buprestidae were recorded: *Agrilus cuprescens* (Ménétriés) *, Agrilus pensus* (Horn) *, Buprestis nutalli* (Kirby) *, Chrysobothris scabripennis* (Gory and Laporte) *, Dicerca pugionata* (Germar). The capture records for the 44 beetle species collected, including the number collected by county and known larval and adult hosts, are provided in Appendix A.

**Figure 2.**
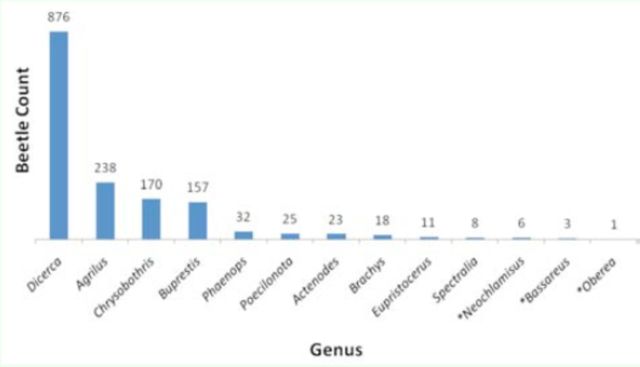
*Cerceris fumipennis* beetle catches by genus in 2009 and 2010 from five colonies in New York State. Nonbuprestid genera are indicated by an asterisk. High quality figures are available online.

Unique (sole record) beetle species were captured in all counties except Onondaga (Three Rivers Wildlife Management Area, Table 2 ). Of the 13 genera captured, the genus *Dicerca* was collected most frequently by *C. fumipennis* (57.3%), almost 4x as often as the second most captured genus, *Agrilus* (15.6%) ( Figure 2 ). The genera *Buprestis* and *Dicerca* contained the largest sized species. In each county, at least half of the catches were from these two genera combined (50–83% of total catches) and overall they accounted for 68% of all beetles caught. The three nonbuprestid genera were infrequently caught (0.6%).

### Phenology

**Figure 3.**
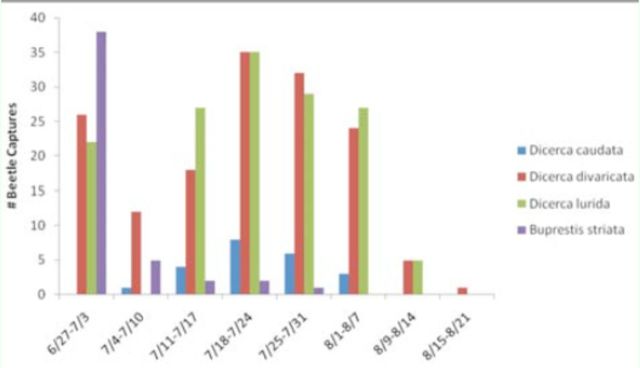
Weekly phenologies of four abundant species of Buprestidae collected from five *Cerceris fumipennis* colonies in New York State in 2010. Sampling began 27 June and ended 21 August. High quality figures are available online.

Many beetle species were collected infrequently across sites. Of the 44 species caught, 20 were collected five or fewer times and 31 species 10 or fewer times. A few beetle species, however, were collected with great frequency. This allowed development of phenologies depicting periods of adult activity for four of these species ( Figure 3 ). The most commonly collected beetle was *Dicerca divaricata* Say (Coleoptera: Buprestidae) (577 records, 37% of catches). Other frequently caught beetles included *Dicerca lurida* F. (205 records, 13%), *Buprestis striata* F. (152 records, 10%), and *Dicerca caudata* Lee (46 records, 3%). Collection of *Dicerca* species peaked in the middle of the sampling period, whereas numbers of *B. striata* declined soon after sampling began. *Dicerca caudata* was caught for the first time in the second week, at a time when numbers of other species encountered during the first week showed a dramatic drop in catches. All except *B. striata* recovered in numbers in the following weeks.

Specimen records from four insect museum collections in New York State were examined for the first and last collection dates for these four most commonly caught species. Collection records revealed a wider range of adult activity for all species. *Dicerca lurida* records ranged from 28 May–29 August, *D. divaricata* from 5 April–16 August, *D. caudata* from unknown to on 5 October (only one record), and *B. striata* from 6 June–6 July.

### Comparison of adjacent sampling sites

**Figure 4.**
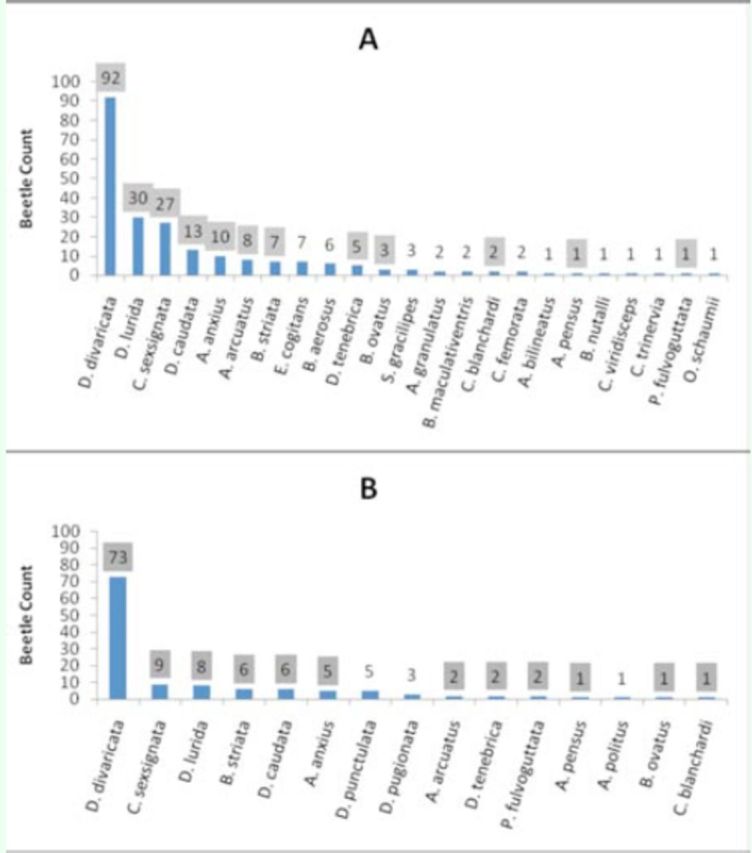
Beetle captures at A) RT9 and B) RT50 *Cerceris fumipennis* colonies in Saratoga County in 2010. Shaded numbers indicate species caught at both sites. High quality figures are available online.

Two wasp colonies, RT9 and RT50, were located approximately 2.7 km apart in Saratoga County. Almost twice the number of beetles were collected at RT9 (n = 226) compared to RT50 (n = 125) in 2010. Composition of beetle catches also differed between the sites. Of the 26 beetle species collected at both sites, 12 were commonly collected from both ( Figure 4 ). RT9 had higher species richness (23 versus 15) and diversity (H’ = 2.23 versus 1.79), and *C. fumipennis* from this colony collected 11 unique beetle species, compared to only three unique species at RT50. Novel species accounted for 11.5% of captures at RT9 and 6.4% at RT50.

### Museum collections

A search of museum records yielded 267 buprestid specimens representing 42 species from the four censused counties (Appendix B). The gross number of beetle species from *C. fumipennis* monitoring was similar to museum collections for counties well-represented in the historical record ( Table 3 ). Museum collections varied in completeness for the different counties, depending on proximity to the sampled county, collection history, and affiliation. Onondaga County, home to the State University of New York College of Environmental Science and Forestry Insect Museum, had the most museum records of all counties. Westchester, Jefferson, and Saratoga Counties did not have resident insect museum collections and these counties were not as well-represented in the historical record.

**Table 3.**
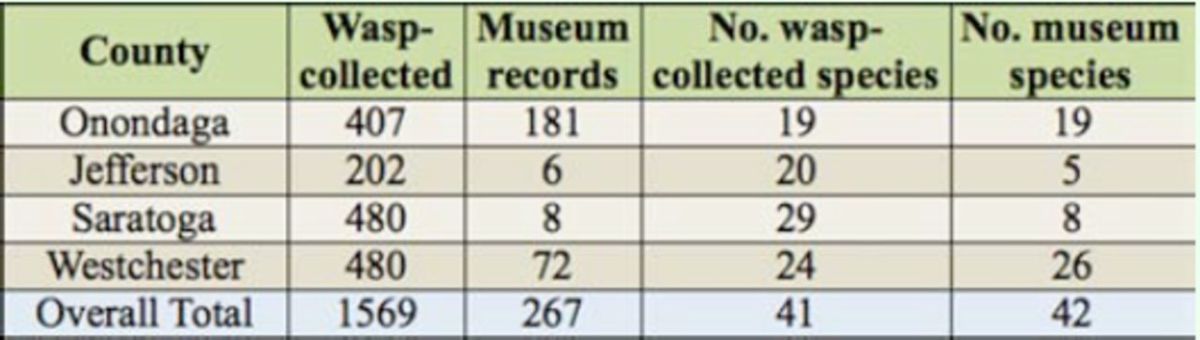
A comparison of buprestids collected from five *Cerceris fumipennis* colonies in New York State in 2009 and 2010 to buprestid collections from four New York State museums. Museum collection dates ranged from 1902 to present.

### Prey range

**Figure 5.**
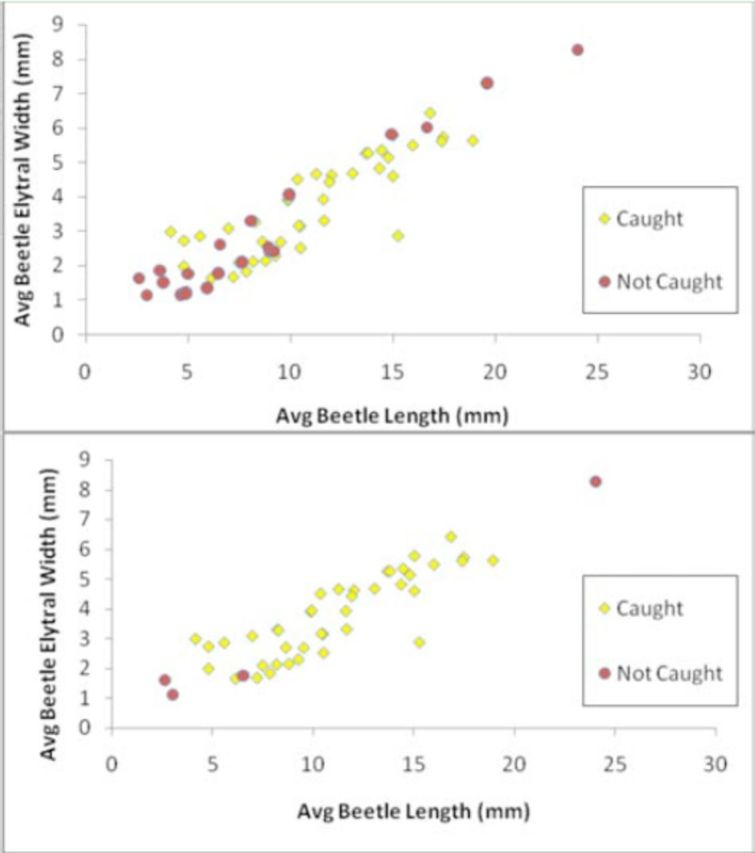
Mean size of beetle species caught by *Cerceris fumipennis* (diamonds) and species that were not caught (circles). Species not caught were compiled by reviewing buprestid collections from four New York State museums. A) Includes all measured beetles. B) After removal of museum specimens whose flight periods do not overlap with the *Cerceris fumipennis* flight period. Note: circles/diamonds overlap for several species. High quality figures are available online.

All beetles caught by *C. fumipennis* fell within a range of 4.1–18.9 mm in length ( Table 4 ). The longest and broadest beetle caught by *C. fumipennis* , *Buprestis maculativentris,* measured 18.9 mm in length and 5.6 mm wide at the thorax (where *C. fumipennis* hold beetles during flight). Mean size measurements of 40 beetle species caught by *C. fumipennis* were plotted along with the 22 species identified in the museum records that were not caught to assess distribution of prey sizes ( Figure 5A ). Six buprestid species from museum collections were outside of the recorded prey size range. The four smallest buprestids ranged from 2.6–3.8 mm long, and the two largest beetles, both in the genus *Chalcophora* , were 19.6 and 24.0 mm long and 0.3–1.3 mm wider than the largest beetle collected from *C. fumipennis* , *Buprestis maculativentris* . The smallest and largest beetles from museum collections that were not caught by *C. fumipennis* fell outside of the 5 ^th^ and 95 ^th^ percentiles for length of all *C. fumipennis* -caught beetles measured. Activity periods of adult buprestids were also taken into account by looking at dates on which uncaught museum specimens were collected. Records of uncaught species indicate that 18 out of 22 species were historically collected outside of the *C. fumipennis* flight, and thus monitoring, period ( Figure 5B ).

**Table 4.**
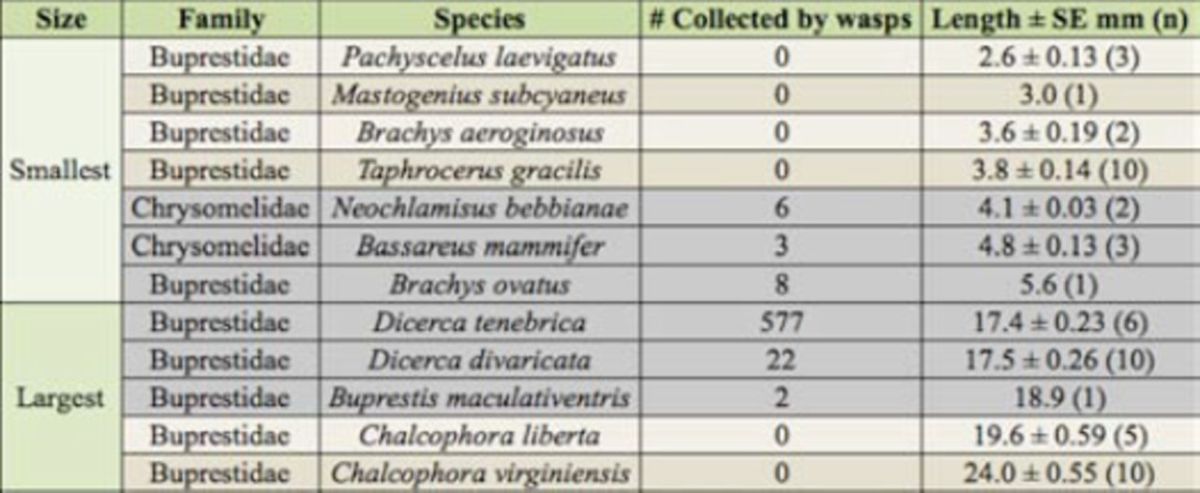
Average lengths of the largest and smallest beetles recorded from collection and museum records in the censused counties. The six species shaded in gray represent the three smallest and three largest beetle species caught by *Cerceris fumipennis* in this study.

### Prey choice

**Figure 6.**
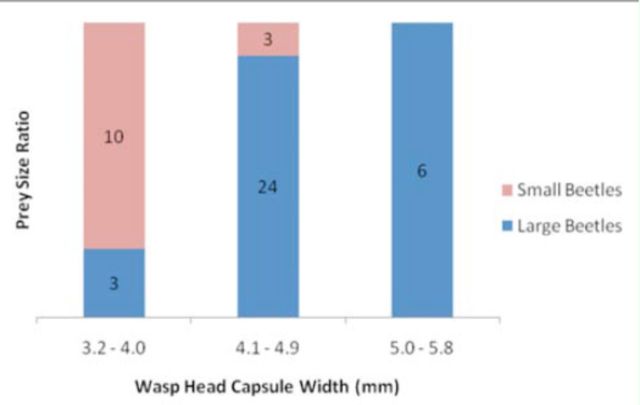
Ratio of large and small beetles captured by three size classes of *Cerceris fumipennis* females. Numbers within bars are numbers of beetles (total n = 46) measured within the different size classes captured by *Cerceris fumipennis* (total n = 46) in each of the *C. fumipennis* size classes. Large beetles were *Buprestis* and *Dicerca* (n = 33), and small beetles were *Agrilus* and *Chrysobothris* (n = 13). Beetle genera were placed into size categories based on average length measurements (see text). High quality figures are available online.

A significant prey size bias was documented for female *C. fumipennis* (G²2,46 = 21.9, *p* < 0.0001) ( Figure 6 ). Larger *C. fumipennis* exclusively caught larger beetles ( *Buprestis* and *Dicerca* spp.), medium *C. fumipennis* collected mostly larger beetles (89%), and though small *C. fumipennis* caught small beetles ( *Agrilus* and *Chrysobothris* spp.), 77% of the time they were also bringing back large ones.

The five largest beetles collected on 22 July 2009 for which biomass was recorded were all *D. divaricata* (mean 216.5 ± 4.9 mg). These large beetles were an order of magnitude heavier than the smaller beetles collected that day, which were from the genera *Agrilus* , *Neochlamisus* , and *Brachys* , with an average weight of 12.7 ± 3.8 mg.

## Discussion

*Cerceris fumipennis* captured more buprestid individuals (1,520) and more species of Buprestidae (41) over these two short sampling seasons than any other single North American study found in an extensive search of the published literature. This survey is part of a larger northeastern biosurveillance project that will be the largest buprestid survey in North America to date (M. Bohne, USDA Forest Service Durham, personal communication). Overall, 16% of species caught in New York were new prey records, and with the inclusion of these seven new records, *C. fumipennis* is now known to prey on > 100 species of beetles. Five of the new prey records were Buprestidae ( *A. cuprescens, A. pensus, B. nutalli, C. scabripennis, D. pugionata* ). *Oberea schaumii* LeConte (Coleoptera: Cerambycidae) is a new record as well and only the second species of this beetle family caught by *C. fumipennis.* This novel prey family has never been recorded for a crabronid wasp prior to 2010 ( Rutledge et al. 2011 ). A new chrysomelid species, *B. mammifer* , was also captured in addition to *Neochlamisus bebbianae* (Brown), a known prey species ( Rutledge et al. 2011 ).

Though much is still unknown about how and why prey are selected, this study hints at some aspects of prey preference. *Dicerca divaricata* , a larger buprestid, was caught most frequently (37% of catches). This beetle uses 11 genera of trees as hosts and is well distributed within the United States ( Nelson 1975 ; Nelson et al. 2008 ). Though potentially common, it is questionable as to whether the high catch frequency accurately reflects abundance at sampling sites or preferential predation. Nevertheless, *C. fumipennis* may prefer this species because of its larger size when compared to most of the other buprestid prey. A majority of species captured (70%) were collected 10 or fewer times and so are either rare in the landscape, less desirable to *C. fumipennis* , or possibly more cryptic (i.e., harder to find).

There was a great deal of variation in beetle species composition by county, as evidenced by the differing catch frequencies. Differences in forest abundance and composition likely affect wood-boring beetle abundance and composition. *Dicerca divaricata* was the most collected beetle in all counties except Jefferson County, where *D. lurida* was caught far more often than *D. divaricata* . One reason may be a greater number of *D. lurida* hosts, i.e., more hickories and oaks. Kurczewski and Miller (1984) documented another New York *C. fumipennis* colony near the town of Auburn where *Agrilus anxius* was caught most frequently followed by *D. lurida* in the apparent absence of *D. divaricata.* This could be an example of prey “switching,” as proposed by Murdoch (1969) , where the most common species is preyed upon with a disproportionately high frequency over a less common species.

From a buprestid sampling standpoint, it would be optimal if available tree species are visited by *C. fumipennis* without preference. The most commonly caught species, *D. divaricata* , uses a diverse array of tree species as hosts (Appendix A). Though most Buprestidae are phloem feeders beneath tree bark, some of the smaller beetles in the tribe Trachyini are exclusively leaf miners. In this study, only two species of Trachyini in the genus *Brachys* , both leafminers of trees, were preyed on by *C. fumipennis.* An examination of museum records revealed other species of Trachyini that mine non-woody plants are present in the censused counties, but these were not collected by *C. fumipennis.* This may reflect a preference by *C. fumipennis* to forage in trees over lower-growing, non-woody vegetation.

Increased host diversity is linked to increased herbivore diversity in other systems ( Gaston 1992 ), and the opposite is also true with increased host abundance being linked to higher numbers of herbivores ( Dempster 1971 ). The results of this study corroborate the tenet that increased tree diversity will result in increased beetle diversity, as the RT9 colony adjacent to the tree nursery with its variety of ornamental trees in Saratoga County had the most beetle species (29) over the two-year study. The highest numbers of conifer dwelling buprestid species (7) were also captured at this colony. This was more than LaFargeville Central School (Jefferson County) and WPR (Westchester County), where there were few or no conifers, based on a perusal of digital images, and the fact that only four conifer feeding species were caught. The second most species rich site was WPR, with 26 beetle species. Using the assumed relationship stated above, the Saratoga site, with higher numbers of conifer and deciduous feeding beetles, likely had the most species rich forest followed by Westchester County. Evaluation of this relationship was beyond the scope of this study, however, vegetative sampling near *C. fumipennis* colonies is strongly encouraged in future studies of this type to elucidate relationships between beetle species diversity and tree diversity.

Weekly sampling of beetles allowed construction of phenologies and provided insights into buprestid life histories as well as *C. fumipennis* foraging behavior. The most puzzling aspect of the phenological trends ( Figure 5 ) was the sudden drop in beetle catches across all sites in the second week. An investigation of weather data excluded poor climactic conditions as a possible explanation as *C. fumipennis* foraging activity drops during cool, windy, cloudy, or rainy days ( Careless 2009 ; W. Hellman, personal observation). Another unexplained phenomenon was the dwindling catches in the last four weeks. Whether beetle numbers decreased, *C. fumipennis* foraging declined, or a combination of both occurred remains to be documented. One possibility is that female *C. fumipennis* were foraging less in order to defend the nest from nest parasites (A. Hook, St. Edwards University, personal communication).

Species composition and capture frequency varied between relatively close colonies as well. RT9 and RT50 were only a few kilo-meters apart; however, beetle species and numbers were dissimilar between the two locations. Of the 26 species caught, only 12 were common to both sites, even though > 88% of catches were from the common species. The fact that both colonies were located on tree farms with various and presumably different ornamental tree species likely factored into these differences.

These data suggest a finite range of prey length for *C. fumipennis* of 4–19 mm, although weight may be equally, if not more, important. Smaller buprestids (< 4 mm) may avoid capture due to *C. fumipennis* being unable to manipulate and carry them or due to their less attractive size. The four smallest beetles measured from museum records were all < 4 mm and are not known prey items of *C. fumipennis* . Smaller beetles can weigh 1/20 ^th^ of the larger beetles, and so it would be inefficient for females to expend the energy to catch them if larger prey were available. Optimal foraging theory (MacArthur and Pianka 1966) would predict a greater payoff by preferentially targeting larger beetles up to a certain threshold where costs exceed reward to reduce the number of foraging flights *C. fumipennis* must take to provision a single cell. Nest excavation reported previously in the literature also supports optimal foraging theory and the idea that biomass influences provisioning. At one site, three relatively larger *Dicerca* beetles were used to provision a single *C. fumipennis* larval cell, while cells containing smaller *Agrilus* contained up to 51 beetles ( Hook and Evans 1991 ).

Prey choice recorded in this study was overwhelmingly in favor of larger beetles ( Figure 6 ). Other sphecoid wasps are known to select prey based on size with a marked preference for larger prey ( Elgar and Jebb 1999 , Polidori et al. 2005 ). In accordance with optimal forage predictions, the majority of prey captured by *C. fumipennis* (66%) were larger beetles from the genera *Dicerca* and *Buprestis* , with > 37% of all captures being *D. divaricata.* According to optimal foraging theory, *D. divaricata* would be a logical prey item, as it is both large (the second largest beetle preyed upon by *C. fumipennis* ) and common (museum records report *D. divaricata* presence in each censused county and provide over 60 unique collection locations from other counties within New York State). Only small *C. fumipennis* preyed frequently on smaller beetles, a trend found in studies of other predatory wasps ( Gwynne and Dodson 1983 ; Polidori et al. 2005 ). The size variation in female *C. fumipennis* may prevent smaller *C. fumipennis* from preying on larger beetles, but it has been suggested that the presence of smaller *C. fumipennis* may reduce intraspecific competition for large prey and this may, over time, promote speciation through niche partitioning ( Stubblefield et al. 1993 ; Swanson et al. 2003 ; Woo et al. 2008 ).

An upper limit to prey size is reasonable, as very large beetles would be too heavy and broad for *C. fumipennis* to handle and transport. Only two *Chalcophora* species that may be too large for predation were identified from museum collections from the censused counties. These beetles were broader (6.0–7.0 mm wide at the thorax) than the broadest of beetles collected from *C. fumipennis* ( *D. maculativentris* , 5.6 mm) *.* It was not possible to weigh live *Chalcophora* , but dried specimens are volumetrically much larger than other prey and are expected to be significantly heavier than *Dicerca* . *Cerceris fumipennis* was observed to strug-gle flying with larger *D. divaricata* , and in some cases it could not take off once it landed.

Another important collection limitation is whether a particular beetle species is active during the *C. fumipennis* flight period. A number of buprestids may emerge too early or late in the season for *C. fumipennis* to catch in New York State. Only four of the 22 species not caught by *C. fumipennis* in New York museum records were noted as being collected during the wasp’s flight period. In New York, the earliest wasps emerged around 27 June and had mostly senesced by 21 August. Beetles whose flight periods do not overlap with these dates would not be available as prey for foraging *C. fumipennis* . Alternatively, it is possible museum collection dates did not provide adequate insights into beetle flight periods, and that these beetles actually do occur during the *C. fumipennis* flight season (and in fact eight of the 22 species noted as being collected outside of the flight period are already known *C. fumipennis* prey items). If so, there may be other factors influencing why beetles were not caught, e.g., rare in the landscape, hosts not visited or not present near the *C. fumipennis* colony.

The factors listed above may influence why the four beetle species that were not excluded by temporal records were not utilized by *C. fumipennis* ( Figure 5B ). *Pachyschelus laevigatus* (Say) (Coleoptera: Buprestidae) and *Mastogenius subcyaneus* (LeConte) were the smallest buprestids measured in this study (2.6 and 3.0 mm long) and are outside of the prey size range documented by this study. *Chalcophora virginiensis* (Drury) was the largest buprestid measured (24.0 mm), and though it is active at the same time as *C. fumipennis* according to museum collection dates, it may be too large to prey upon. The only remaining beetle is *Agrilus ruficollis,* which was not collected in this study but is a documented prey item of *C. fumipennis* ( Scullen 1965 ; Evans 1971 ). This beetle may either be rare in the landscape or its hosts may not be present near the censused colonies.

Twelve genera of Buprestidae drawn from all four buprestid subfamilies are represented in the known prey records of *C. fumipennis* ( Careless 2009 ). In this study alone, three of the four North American buprestid subfamilies and 10 genera were represented. Nelson et al. (2008) lists 52 buprestid genera known in the United States and Canada, but only 20 have been reported in New York. Of the eight genera that at present are not known to be preyed on by *C. fumipennis* , only four ( *Taphrocerus, Pachyscelus, Mastogenus, Chalcophora* ) were present in museum collection records from censused counties, and representatives of all of these species were outside of the *C. fumipennis* prey size range. This means *C. fumipennis* exhibited a sampling efficiency of approximately 75% across available buprestid genera in New York. Increased sampling efforts may result in the capture of the remaining four genera ( *Agrilaxia, Xenorhipis, Melanophila, Par-agrilus* ); however, the factors discussed above may also impact whether a particular beetle species is captured or not. From a strictly practical viewpoint, beetles in the genus *Agrilus* tend to be the most economically important and, fortunately, they are readily detectable by *C. fumipennis* sampling.

This study provides information critical to using *C. fumipennis* as a sampling tool for Buprestidae. Other critical information necessary is a preliminary understanding of the *C. fumipennis* foraging range. It is suggested that prey-host records may be useful for approximating a true maximum forage distance. Many buprestids use a single or limited number of hosts, some of which are rare in some landscapes. Measurement from a *C. fumipennis* colony to a beetle’s host tree would provide a strong inferential reference point to estimate maximum foraging distances. It is also the case that colonies are not always present by an intended sampling site. Because of this, research into developing protocols for translocating *C. fumipennis* is critical.

This research supports use of *C. fumipennis* as an effective and efficient tool for sampling Buprestidae. Sampling does not require extensive training or expensive equipment, and because *C. fumipennis* are not known to sting, there is no associated danger. They can be used to sample buprestid populations, conduct natural history surveys, and detect invasive species.

## Figures and Tables

**Appendix A.**
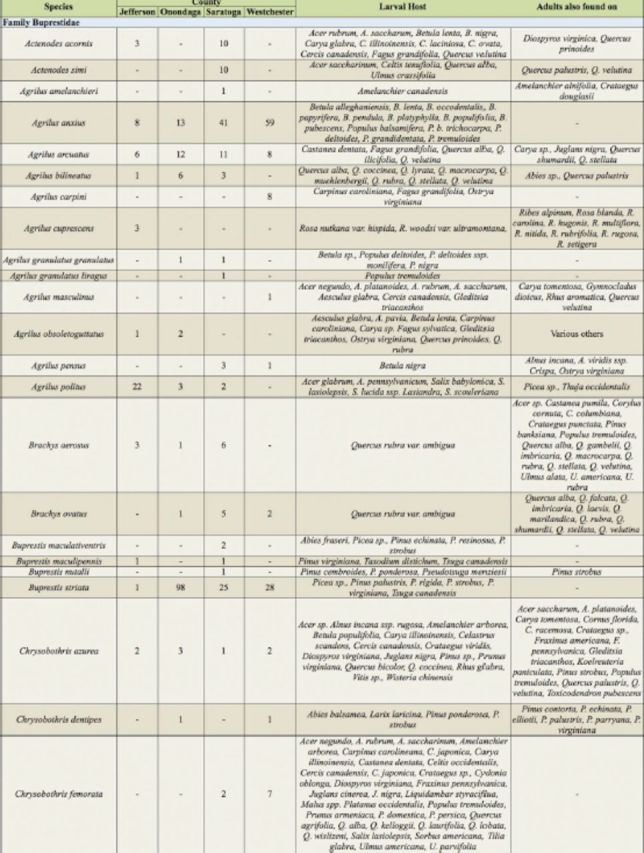

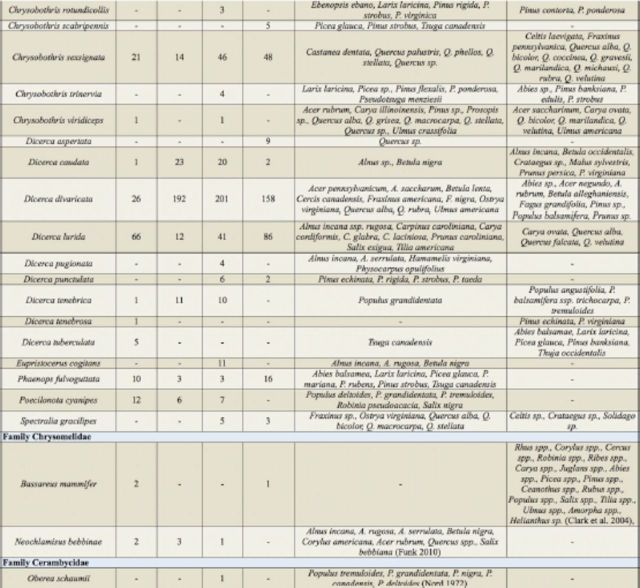
Comprehensive list of *Cerceris fumipennis* prey items by county. Adult and larval host plant information is provided for each species. Host information compiled from Nelson et al. 2008 unless otherwise noted.

**Appendix B.**
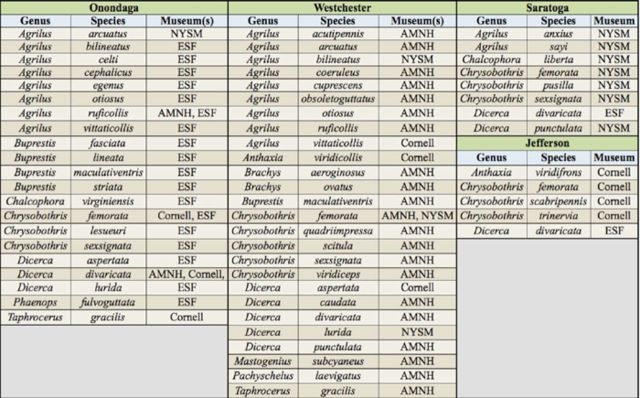
Buprestidae museum records from four New York collections.

NYSM = New York State Museum, ESF = State University of New York College of Environmental Science and Forestry, AMNH = American Museum of Natural History, Cornell = Cornell University

## Abbreviations

RT9: Route 9
RT50: Route 50
WPR: Ward Pound Ridge County Park
